# Rat limbal niche cells can induce transdifferentiation of oral mucosal epithelial cells into corneal epithelial-like cells in vitro

**DOI:** 10.1186/s13287-018-0996-9

**Published:** 2018-09-26

**Authors:** Xin-Yue Zhao, Hua-Tao Xie, Chao-Ye Duan, Jing Li, Ming-Chang Zhang

**Affiliations:** 1 Department of Ophthalmology, Union Hospital, Tongji Medical College, Huazhong University of Science and Technology, Wuhan, 430022 China; 2 Department of Ophthalmology, Tongji Hospital, Tongji Medical College, Huazhong University of Science and Technology, Wuhan, 430022 China

**Keywords:** Limbal niche cells, Oral mucosal epithelial cells, Cell transdifferentiation, Limbal stem cell deficiency, Ocular reconstruction

## Abstract

**Background:**

Cultivated oral mucosal epithelial cells (OMECs) are widely used in the treatment of limbal stem cell deficiency (LSCD) for their ocular reconstruction capability. As the most important component of the limbal microenvironment, limbal niche cells (LNCs) play a key role in the direction of stem cell differentiation. In this study, we investigated whether LNCs can induce the transdifferentiation of rat OMECs to corneal epithelial-like cells.

**Methods:**

We isolated OMECs and LNCs from rats by dispase and collagenase, respectively, to establish a three-dimensional or Transwell coculturing system. NIH-3T3 cells and renewed LNCs were also used as feeder layers in the Transwell system to compare their ability to support the OMECs. The airlift method was used for the culture of OMECs to obtain a stratified epithelial sheet. Cocultured OMECs were characterized by reverse-transcription polymerase chain reaction, Western blotting, hematoxylin and eosin staining, and immunohistochemistry.

**Results:**

The cocultured OMECs showed corneal epithelial-like morphology and expressed the corneal epithelial markers CK12 and Pax6 in most cocultured systems. Furthermore, we found that the expression level of CK12, Pax6, and proliferation marker Ki67 was upregulated when compared with that of other groups by renewing the LNCs in the Transwell system (*p* < 0.05, *n* = 3), suggesting that this might be a potential method for improving the efficiency of transdifferentiation. The obtained stratified epithelial sheet expressed CK3 and CK12.

**Conclusion:**

Through coculturing OMECs and LNCs in vitro, we successfully cultivated corneal epithelial-like OMECs. This investigation is of great significance for the treatment of LSCD and ocular surface reconstruction.

**Electronic supplementary material:**

The online version of this article (10.1186/s13287-018-0996-9) contains supplementary material, which is available to authorized users.

## Background

Limbal stem cell deficiency (LSCD) is the main cause of most blinding keratopathies [1]. Autologous limbal stem cell transplantation is important for ocular reconstruction [2, 3], and the results of long-term follow-up are very encouraging [4]. However, the use of this method poses a threat to the healthy eye [5], and most serious ocular diseases are binocular, forcing ophthalmologists to select cornea or stem cells of allogeneic origin [6]. Nonetheless, the severe lack of corneal donors in China and the risk of rejection after transplantation restricts allogeneic transplant [7, 8]. Nowadays, cultivated oral mucosal epithelial cell (OMEC) transplantation has shown encouraging results in reconstructing the ocular surface affected by LSCD [9–12]. NIH-3T3 cells are widely used to coculture the oral epithelium for maintenance of undifferentiated epithelial stem cells during amplification [9, 10, 12]. However, this poses a risk of transmitting murine-derived diseases. Moreover, long-term outcomes are less satisfactory; specifically, a high rate of peripheral corneal neovascularization has been observed [13]. Therefore, in recent years, scholars have used other fibroblasts to expand oral mucosal epithelium and corneal epithelium in vitro [11, 14].

Some researchers have reported successful application of the limbal microenvironment in vitro to stimulate embryonic stem cells and adult stem cells to transdifferentiate into corneal-like epithelium [15–19]. As the most important component of the limbal microenvironment, limbal niche cells (LNCs) play a key role in the prevention of differentiation of limbal stem cells [20–22]. Oral epithelial cells and corneal epithelial cells originate from the same ectoderm; however, the only study that attempted to coculture OMECs and LNCs in vitro obtained unsatisfactory results [23]. In this study, we cocultured OMECs and LNCs with a new in-vitro model to observe whether LNCs can induce the transdifferentiation of rat OMECs to corneal epithelial-like cells.

## Methods

### Animals

Sprague–Dawley rats weighing 150–200 g each were supplied by the Animal Research Committee of the Huazhong University of Science and Technology (Wuhan, China). The study was conducted after obtaining approval from the Institutional Animal Care and Use Committee at Tongji Medical College, Huazhong University of Science and Technology (IACUC number S760, 9 September 2016; Additional file 1).

### Isolation of rat central cornea, limbus, and OMECs

**Fig. 1:**
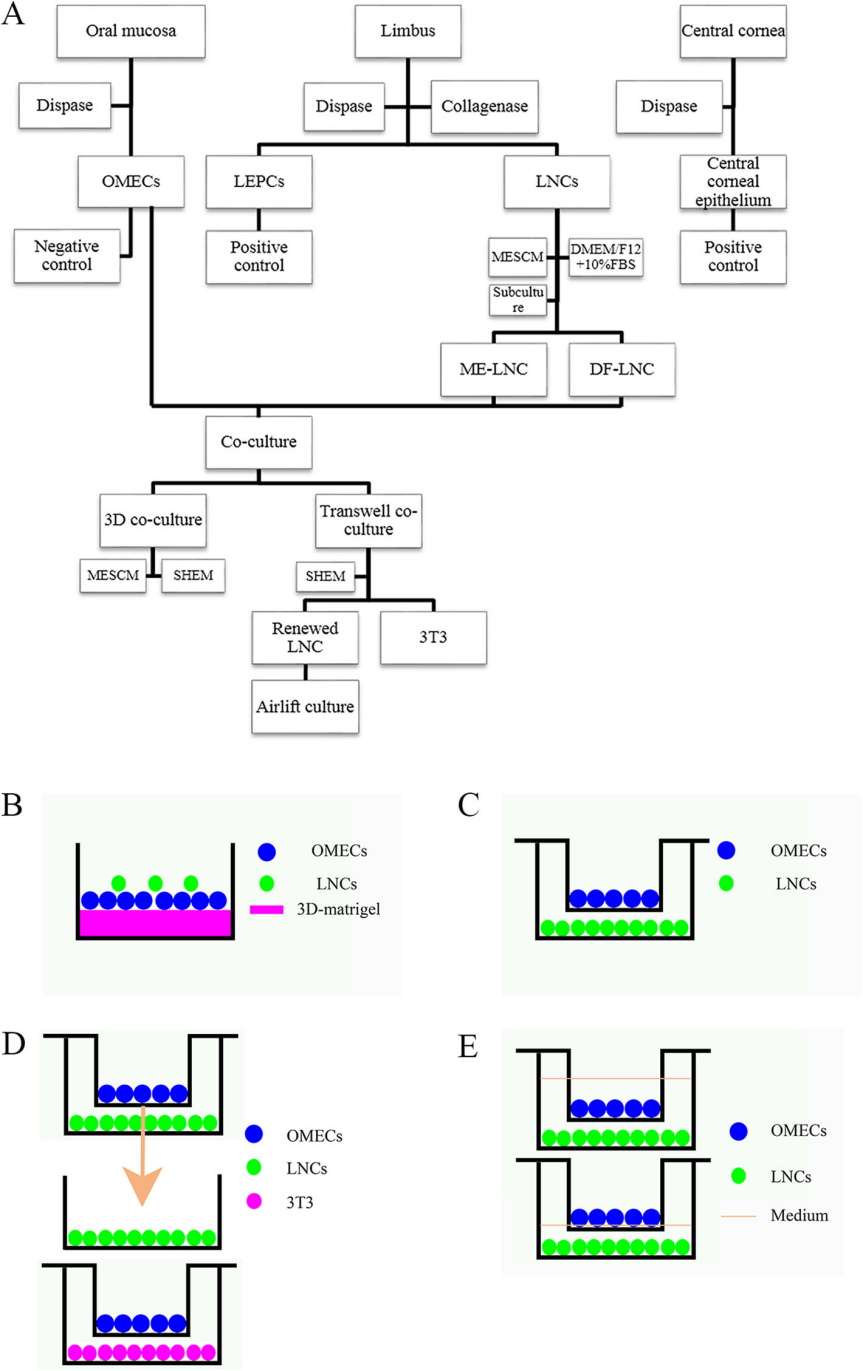
Isolation and coculturing of OMECs and LNCs. **a** Isolation of central corneal epithelial cells, limbal epithelial progenitor cells, limbal niche cells (LNCs), and oral mucosal epithelial cells (OMECs). **b** Three-dimensional coculturing system, **c** Transwell coculturing system, **d** renewed Transwell coculturing system, and **e** Airlift coculturing system. DF-LNC LNCs grown in DMEM/F12 supplemented with 10% FBS, DMEM/F12 Dulbecco’s modified Eagle’s medium, FBS fetal bovine serum, LEPC limbal epithelial progenitor cell, ME-LNCs LNCs grown in MESCM, MESCM modified embryonic stem cell medium, SHEM supplemented hormonal epithelial medium

Isolation of rat central cornea was conducted with a protocol we have reported previously [21]. Briefly, after anesthesia and dislocation of the spinal column in the rats, the eyeball was removed, followed by ring-cutting of the cornea at 1 mm outside the limbus of the cornea. A corneal trephine of 3 mm diameter was used to separate the central cornea and limbus. A portion of the tissue was embedded in paraffin for immunofluorescence, while the other portion was washed three times with phosphate-buffered saline (PBS; Hyclone, Logan, UT, USA), cut into small pieces followed by removal of the iris and endothelium, and digested with 10 mg/mL dispase II (Roche, Indianapolis, IN, USA) for 30 min. Both central and limbal epithelium were separated from the corneal stroma by ophthalmic micro-tweezers. Isolation of OMECs was performed using a previously reported method [24]. In brief, the buccal mucosa was removed for the experiment; a portion was embedded in paraffin for immunofluorescence, while the rest was cut into small pieces and washed three times with PBS. The samples were then digested in 10 mg/mL dispase II for 30 min. OMECs were also separated by ophthalmic micro-tweezers. A flow chart outlining the steps involved in isolation is presented as Fig. 1a.

### Preparation of Matrigel

The Matrigel™ Basement Membrane Matrix (BD Biosciences, San Jose, CA, USA) was prepared in coating or three-dimensional (3D) form following previous reports [20]. Briefly, Matrigel was diluted at a ratio of 1:20 or 1:1 with Dulbecco’s modified Eagle’s medium (DMEM/F12, [1:1]; Hyclone) for coating or 3D, respectively. The diluted Matrigel was then added into the culture plate at 50 μL/cm^2^ or 200 μL/cm^2^ for coating or 3D culture, respectively, followed by incubation at 37 °C with 5% CO_2_ for 1 h. Before cell seeding, the Matrigel coating was removed. All operations were performed in an ice bath.

### Culturing of LNCs

LNC culture was conducted as described previously [20], the steps of which are also included in Fig. 1a. Briefly, the limbus was washed with PBS three times and digested in 1 mg/mL Collagenase A (Sigma-Aldrich, St. Louis, MO, USA) for 3 h. The outcome of digestion was then transferred to an Eppendorf tube for trypsinization in 0.25% trypsin/0.02% ethylenediaminetetraacetic acid (T/E) for 10 min. After neutralization, the samples were centrifuged and resuspended in modified embryonic stem cell medium (MESCM), which consisted of DMEM/F12 supplemented with 10% knockout serum, 5 μg/mL insulin, 5 μg/mL transferrin, 5 ng/mL sodium selenite, 4 ng/mL basic fibroblast growth factor, 10 ng/mL human leukemia inhibitory factor, 50 μg/mL gentamicin, and 1.25 μg/mL amphotericin B. The cells were cultured in six-well plastic culture plates coated with 5% Matrigel. Once confluent, they were passaged at a ratio of 1:4 until passage 3. In our studies, we also used DMEM/F12 supplemented with 10% fetal bovine serum to culture LNCs. The obtained LNCs in MESCM or DMEM/F12 supplemented with 10% fetal bovine serum were designated ME-LNCs or DF-LNCs. The expression level of both the mesenchymal stem cell marker Sox2 and Oct4 of P3 LNCs were examined by reverse-transcription polymerase chain reaction (RT-PCR) and Western blotting, and N-cadherin was examined by immunofluorescence. Double immunofluorescence of Vimentin (Vim) and CK12, ΔNp63α, or Pax6 was also evaluated to verify that purified LNCs were obtained.

### Three-dimensional coculturing of OMECs and LNCs under different conditions

Three-dimensional coculturing of OMECs and LNCs was conducted as shown in Fig. 1b. Briefly, the isolated oral mucosal epithelium was trypsinized by T/E for 15 min. Afterwards, the samples were neutralized, followed by centrifugation and resuspension in MESCM or supplemented hormonal epithelial medium (SHEM), which consisted of DMEM/F12 (1:1) supplemented with 10% fetal bovine serum, 5 μg/mL insulin, 5 μg/mL transferrin, 5 ng/mL sodium selenite, 0.45 μg/mL hydrocortisone, 20 ng/mL epidermal growth factor, 10 ng/mL human leukemia inhibitory factor, and 100 U/mL penicillin and streptomycin. Cell counting confirmed that the OMECs and P3 LNCs were mixed at a ratio of 4:1. Then, the mixture was seeded at a density of 12 × 10^4^ cells/cm^2^ for OMECs and 4 × 10^4^ cells/cm^2^ for LNCs in 24-well plastic culture dishes that contained 3D Matrigel. According to LNCs and the media used in the 3D coculturing system, we established four different experimental groups, including ME-ME (ME-LNCs, MESCM for 3D coculturing), ME-SH (ME-LNCs, SHEM for 3D coculturing), DF-ME (DF-LNCs, MESCM for 3D coculturing), and DF-SH (DF-LNCs, SHEM for 3D coculturing). Furthermore, the primary OMECs and central corneal epithelium were considered as controls. The culture medium was changed every 2 days. The expression level of the specific corneal markers CK12 and Pax6 in the cocultured OMECs and LNCs was observed. On day 7 of coculturing, 10 mg/mL dispase II was added to each group to dissolve the Matrigel at 37 °C with 5% CO_2_ for 2 h. A portion of the resulting samples was prepared for RT-PCR. The rest were used for immunofluorescence after trypsinization to suspend the cells. The positive rates of CK12 and Pax6 expression were determined by counting in three randomly selected 400-fold fields of vision and calculating the average positive rate; the experiment was repeated three times.

### Transwell coculturing of OMECs and LNCs

We also attempted to coculture OMECs and LNCs in a Transwell system to obtain a transplantable epithelial sheet using the model provided in Fig. 1c. When the P3 ME-LNCs and DF-LNCs reached confluence, they were treated with 4 μg/mL mitomycin C for 2 h to restrain their growth. After washing with PBS three times, they were ready for coculturing. The Transwell inserts (0.4 μm, polyethylene terephthalate; Corning Inc., Corning, NY, USA) coated with 5% Matrigel were placed in the wells. The suspended OMECs were seeded at a density of 5 × 10^4^/cm^2^ in the insert. SHEM (1.5 mL and 2.6 mL) was added to the upper and lower chamber, respectively, according to the Transwell instructions. According to the medium used for LNC culture in the Transwell system, we established two experimental groups, including ME (Cocultured OMECs supported by ME-LNCs in Transwell) and DF (Cocultured OMECs supported by DF-LNCs in Transwell). Aside from 3D coculturing, we also cultured OMECs and limbal epithelial progenitor cells (LEPCs) by the suspension method on 5% Matrigel-coated plastic without the support of LNCs as a control. The medium was changed every 2 days. The expression level of the specific corneal markers CK12 and Pax6 in the cocultured OMECs was also observed. On day 10–14 of coculturing in the Transwell system, we used T/E to digest the OMECs for further RT-PCR and Western blot assay. Furthermore, we placed 5% Matrigel-coated slides into the insert in advance for immunofluorescence staining to detect expression of CK3, CK12, Pax6, and ΔNp63α in ME and DF.

### Renewing LNCs and comparison with 3T3 cells in the Transwell system

After successful coculturing of OMECs and LNCs in the Transwell system, we observed the ability of LNCs to support the growth and maintain the phenotype of OMECs compared with that of the gold-standard cell line NIH-3T3 (ATCC, Manassas, VA, USA). At the same time, we observed whether renewing the LNCs in the Transwell system could influence the phenotype of the OMECs. NIH-3T3 cells were also treated with 4 μg/mL mitomycin C for 2 h to restrain their growth and served as a feeder layer similarly to LNCs as we described above. LNCs were renewed by passaging at different ratios in advance, with subsequent steps as shown in Fig. 1d. Briefly, the P2 confluent LNCs were passaged at ratios of 1:4, 1:8, and 1:16. When we changed the medium, we moved the insert to the well containing LNCs, which had been passaged at 1:8. In the same way, we moved the insert to the well containing LNCs that had been passaged at 1:16 when we changed the medium the next time. LNCs were renewed at least twice before cocultured OMECs reached confluence. This renewing group was designated DF+. The cornea epithelium was used as a probable positive control. The expression level of the specific corneal markers CK12 and Pax6, proliferation marker Ki67, and stem cell marker ΔNp63α in the cocultured OMECs was observed. On day 10–14, the cocultured cells were trypsinized by T/E for further RT-PCR and Western blotting. All materials used in the isolation and cell culture are included in Additional file 1: File S1.

### Airlift culture

The confluent OMECs cocultured with renewed DF-LNCs in Transwell were cultured for an additional 2 weeks by lowering the compartment containing the medium to the bottom of the insert (Fig. 1e). Cocultured OMECs was embedded in paraffin for hematoxylin and eosin staining and immunofluorescence.

### RT-PCR

RT-PCR was performed following a standard protocol [24]. Briefly, 1 mL Trizol (Aidlab, Beijing, China) was used to extract total RNA. The RNA was then synthesized by DNase and Hiscript Reverse Transcriptase to synthesize cDNA (RNase H, GeneCopoeia, Rockville, MD, USA). β-actin primers were used as an internal control. The PCR amplification was performed under the following conditions: denaturation at 50 °C for 2 min and 95 °C for 10 min, followed by 40 cycles at 95 °C for 30 s and 60 °C for 30 s. All RT-PCR experiments were performed in triplicate for each group. The primer sequences used in RT-PCR are listed in Additional file 1: File S2.

### Western blotting

Western blotting was performed following a standard method that has been previously reported [25]. Briefly, the protein concentration was determined using the bicinchoninic acid protein assay (Beyotime, Shanghai, China). The same amount of protein in the total cell extract was isolated by 10% sodium dodecyl sulfate-polyacrylamide gel electrophoresis and transferred to a polyvinylidene fluoride (PVDF) membrane, which was then blocked with 5% (wt/vol) fat-free milk. The membrane was continuously incubated with specific primary antibodies and their respective secondary antibodies, with β-actin or GADPH as a loading control. The immunoreactive bands were detected by chemiluminescent reagents. The results were scanned by BandScan to analyze the grayscale values of the film.

### Hematoxylin and eosin staining

The slides were first stained in Harris’ hematoxylin for 8 min. After washing, they were immersed in 1% acid and then 1% ammonia for differentiation. The slides were rinsed in water for 1 h and then rinsed once in distilled water, followed by treatment with 70% and 90% alcohol, respectively, for 10 min for dehydration. They were then stained in eosin-phloxine solution for 2 to 3 min, dried, and observed under a microscope.

### Immunohistochemistry

After being immersed in PBS three times, the slides were treated with 0.5% Triton X-100 at room temperature, and serum was used to block the antigen. The cells were then incubated with primary and secondary antibodies and subsequently stained with DAPI. Images were acquired with a fluorescence microscope (BX53, Olympus, Tokyo, Japan). All antibodies used in the experiment are included in Additional file 1: File S3.

### Statistical analysis

Data in the figures are shown as the mean ± SD. At first, one-way analysis of variance (ANOVA) was used to compare the general differences, followed by paired Student’s *t* test if *p* < 0.05 to compare two groups for RT-PCR and Western blot experiments. Unpaired Student’s *t* test was used to compare the positive cell rate. *p* < 0.05 was considered statistically significant.

## Results

### Immunofluorescence of the rat oral mucosa, central cornea, and limbus tissues

**Fig. 2:**
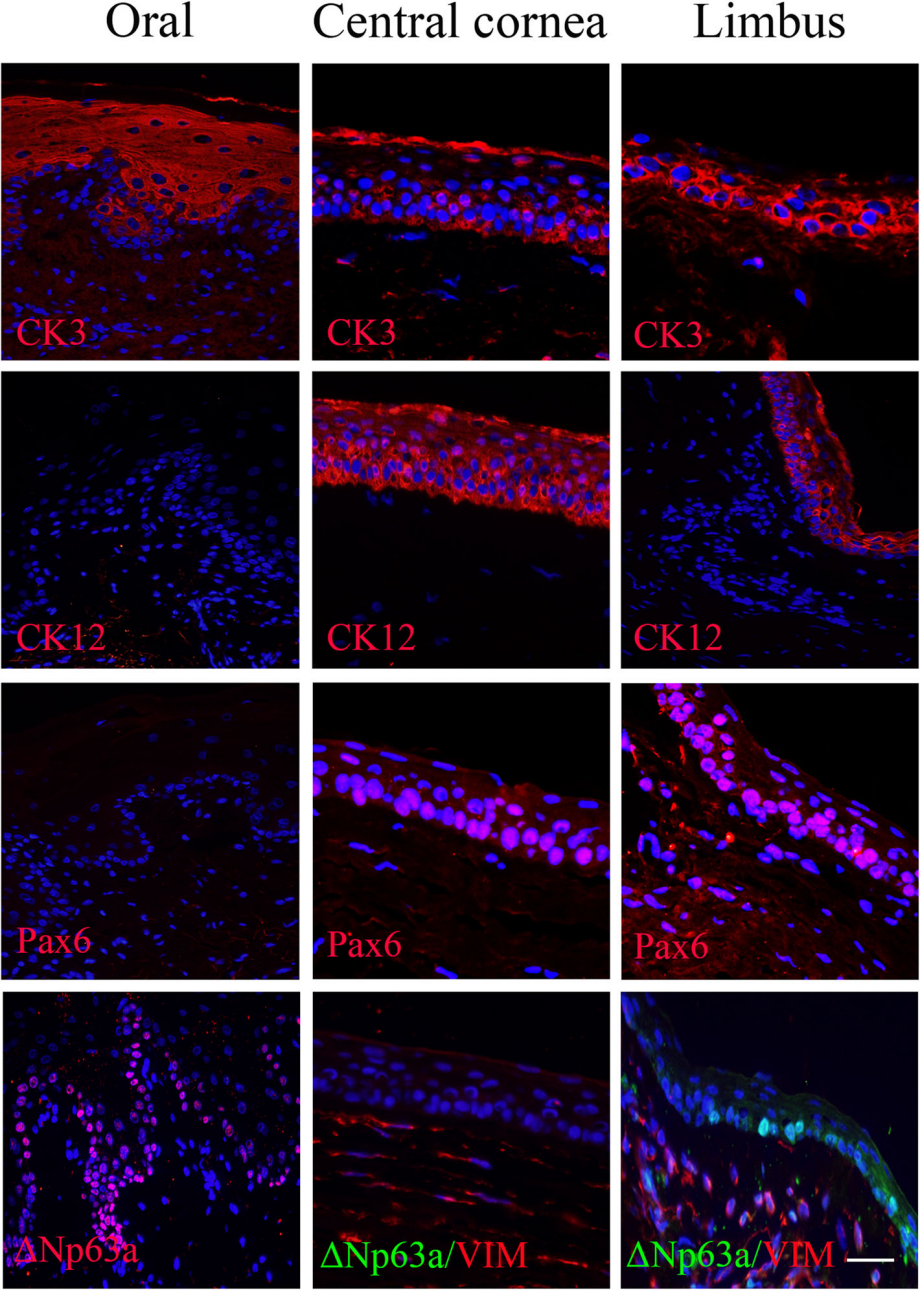
Immunofluorescence of oral mucosa, central cornea, and limbus. Rat oral mucosa, central cornea, and limbus were single-stained for CK3, CK12, Pax6, or ΔNp63α (red fluorescence) or double-stained for ΔNp63α (green fluorescence) and Vim (red fluorescence). Nuclei were stained by DAPI. Scale bar = 100 μm

The oral mucosa, central cornea, and limbus all expressed CK3 over the entire epithelial layers. However, the oral mucosa did not express CK12 or Pax6, while the central cornea and limbus expressed CK12 and Pax6 over the whole epithelium. Furthermore, the oral mucosa and limbus both expressed ΔNp63α at the basal layer of the epithelium, whereas the central cornea did not express ΔNp63α (Fig. 2).

### Molecular phenotype characterization of LNCs

**Fig. 3:**
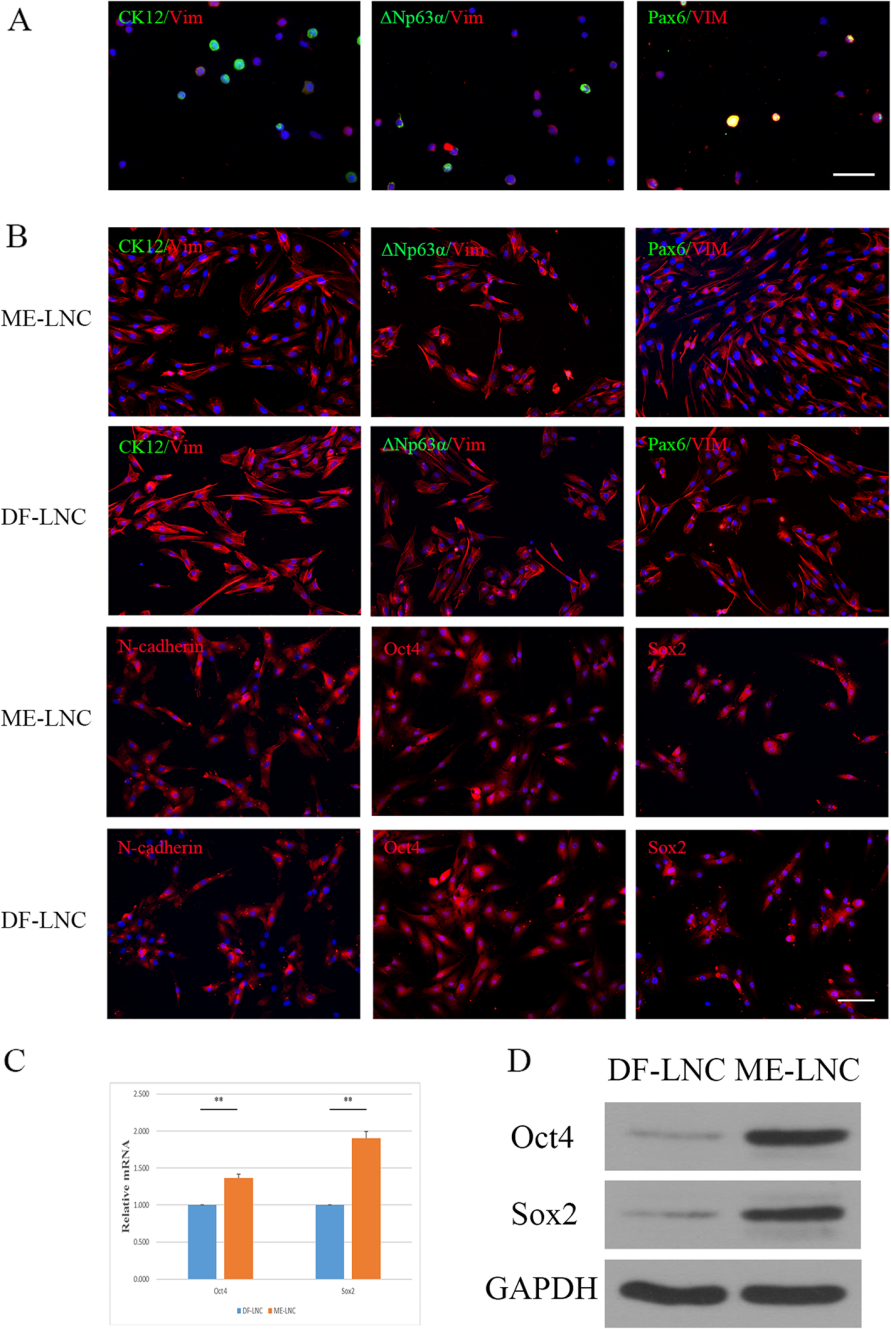
Molecular phenotype characterization of LNCs. **a** P0 limbal niche cells (LNCs) were double-stained for Vim (red fluorescence) and CK12 (green fluorescence), ΔNp63α (green fluorescence) or Pax6 (green fluorescence). **b** P3 LNCs grown in MESCM (ME-LNCs) and LNCs grown in DMEM/F12 + 10% FBS (DF-LNCs) were double-stained for Vim (red fluorescence) and CK12 (green fluorescence), ΔNp63α (green fluorescence) or Pax6 (green fluorescence). Furthermore, they were single-stained for N-cadherin, Oct4, or Sox2 (scale bar = 100 μm). **c** Relative mRNA levels of Oct4 and Sox2 expressed in P3 DF-LNCs and ME-LNCs. **d** Western blot analysis of Oct4 and Sox2 expression in P3 DF-LNCs and ME-LNCs; GAPDH was used as a loading control. **p < 0.01

P0 LNCs were double-stained for Vim and CK12, ΔNp63α or Pax6 in either MESCM or DMEM/F12 supplemented with 10% fetal bovine serum. The P0 LNCs included both CK12^+^, ΔNp63α^+^, Pax6^*+*^ and Vim^+^ cells (Fig. 3a). Double immunofluorescence of Vim and CK12, ΔNp63α or Pax6 in P3 ME-LNCs and DF-LNCs was also assessed to confirm that purified LNCs were obtained from rats. Both P3 ME-LNCs and DF-LNCs were CK12^–^, ΔNp63α^–^, Pax6^–^, Vim^+^, N-cadherin^+^, Oct4^+^, and Sox2^+^, indicating that they had been purified and represented the phenotype of limbal niche cells (Fig. 3b). RT-PCR and Western blot were performed to compare the expression levels of Oct4 and Sox2 between ME-LNCs and DF-LNCs. The expression levels of Oct4 and Sox2 in ME-LNCs were significantly higher than that in DF-LNCs. The relative mRNA level of Oct4 was 1.363 ± 0.054-fold for ME-LNCs compared with DF-LNCs (*p* = 0.00318). The relative mRNA level of Sox2 was 1.904 ± 0.089-fold for ME-LNCs compared with DF-LNCs (*p* = 0.00735) (Fig. 3c). Western blotting showed that ME-LNCs expressed higher Oct4 and Sox2 than DF-LNCs at the protein level. Detailed data are provided in Additional file 1: Files S4 and S5.

### Transdifferentiation of OMECs into corneal epithelial-like cells under 3D coculturing conditions

**Fig. 4:**
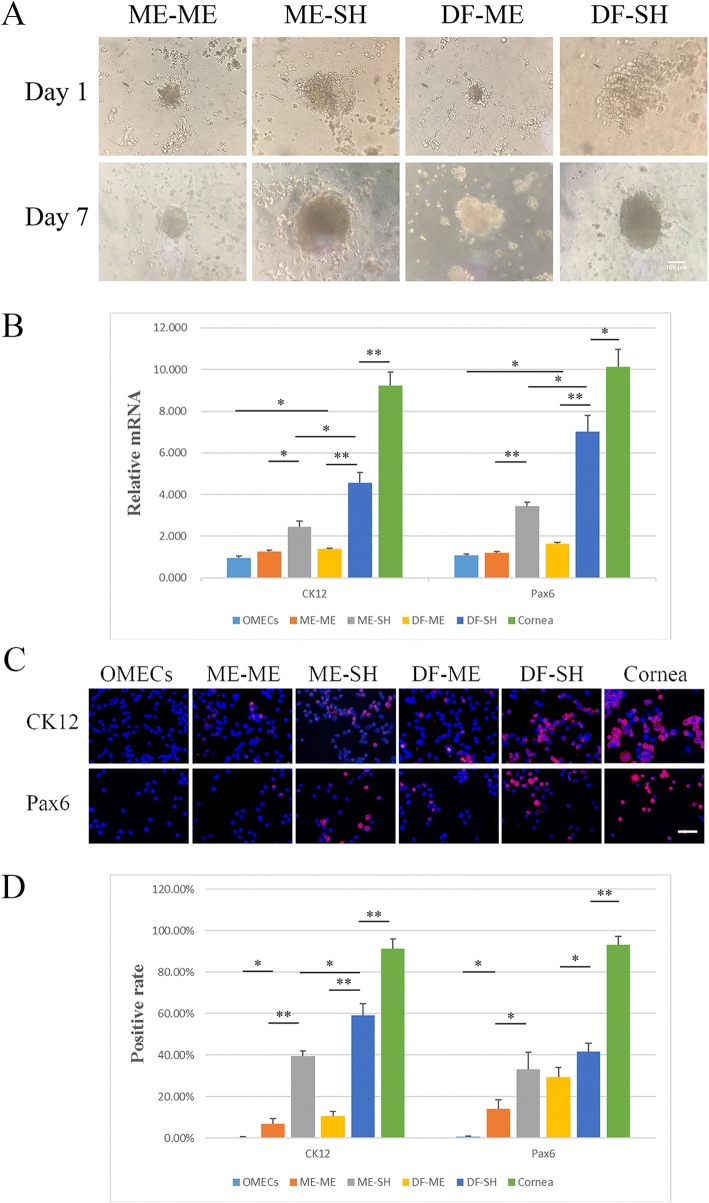
Three-dimensional coculturing of oral mucosal epithelial cells (OMECs) and limbal niche cells (LNCs) under different conditions. **a** Morphologic characterization of OMECs and LNCs cocultured on 3D Matrigel. **b** The relative mRNA levels of CK12 and Pax6 in cocultured OMECs and LNCs. **c** The immunofluorescence of suspended OMECs and LNCs cocultured on 3D Matrigel. Cells were single-stained for CK12 or Pax6 (red fluorescence), and nuclei were stained by DAPI. Scale bar = 100 μm. **d** The positive rate of CK12 and Pax6 expression in cocultured OMECs and LNCs. **p < 0.01, *p < 0.05. DF-ME LNCs grown in DMEM/F12 (DF-LNCs) 3D cocultured in MESCM, DF-SH DF-LNCs 3D cocultured in SHEM, ME-ME LNCs grown in MESCM (ME-LNCs) 3D cocultured in MESCM, ME-SH ME-LNCs 3D cocultured in SHEM

OMECs and LNCs grown on the 3D Matrigel began to form spheres on the first day of coculturing and the spheres became rounder and bigger by day 7 (Fig. 4a), similarly to limbal stem cells and LNCs. We observed that the groups involving SHEM generated a larger sphere than those involving MESCM. Groups involving MESCM generated smaller and rounder spheres. RT-PCR showed that the expression level of CK12 in OMECs, ME-ME, ME-SH, DF-ME, DF-SH, and the cornea was 0.963 ± 0.092, 1.267 ± 0.058, 2.452 ± 0.262, 1.395 ± 0.042, 4.572 ± 0.475, and 9.216 ± 0.670, respectively. No statistically significant difference between OMECs and ME-ME was observed (*p* = 0.06837, *n* = 3), but OMECs were statistically different from the other groups (*p* < 0.05, *n* = 3). When ME-ME was compared with ME-SH, a statistically significant difference was noted (*p* = 0.01461, *n* = 3), while DF-ME and DF-SH had similar results (*p* = 0.00871, *n* = 3). The expression level of CK12 in DF-SH was also upregulated when compared with that of ME-SH (*p* = 0.03653, *n* = 3). In addition, the DF-SH group, which expressed the highest level of CK12, was statistically different from the cornea group (*p* = 0.00073, *n* = 3). The expression level of Pax6 in OMECs, ME-ME, ME-SH, DF-ME, DF-SH, and the cornea was 1.071 ± 0.072, 1.206 ± 0.059, 3.440 ± 0.189, 1.625 ± 0.088, 7.031 ± 0.782, and 10.143 ± 0.830, respectively. OMECs were statistically different from the other groups (*p* < 0.05, *n* = 3), except for ME-ME (*p* = 0.21115, *n* = 3). Furthermore, when comparing ME-ME with ME-SH and DF-ME with DF-SH, statistical differences were observed (*p* = 0.00386 for ME-ME and ME-SH, *p* = 0.00756 for DF-ME and DF-SH, *n* = 3). The expression level of Pax6 in DF-SH was also upregulated when compared with that in ME-SH (*p* = 0.01067, *n* = 3), whereas the expression level in the DF-SH group did not reach that in the cornea (*p* = 0.01258, *n* = 3). Results are shown in Fig. 4b, and detailed data are included in Additional file 1: File S6. The immunofluorescence results showed that OMECs were largely negative for CK12 and Pax6 expression, while the cornea epithelium was positive for CK12 and Pax6 expression (Fig. 4c). Positive cell counting revealed results very similar to those of RT-PCR, except that ME-ME was statistically different from OMECs regarding the positive rate for both CK12 and Pax6 (*p* = 0.04075 for CK12, *p* = 0.03035 for Pax6, *n* = 3). Furthermore, no statistically significant difference between ME-SH and DF-SH in the positive rate of Pax6 was observed (*p* = 0.21083, *n* = 3). Results for CK12 and Pax6 positivity are shown in Fig. 4d and detailed data can be found in Additional file 1: File S7.

### Transwell coculturing of OMECs and LNCs

**Fig. 5:**
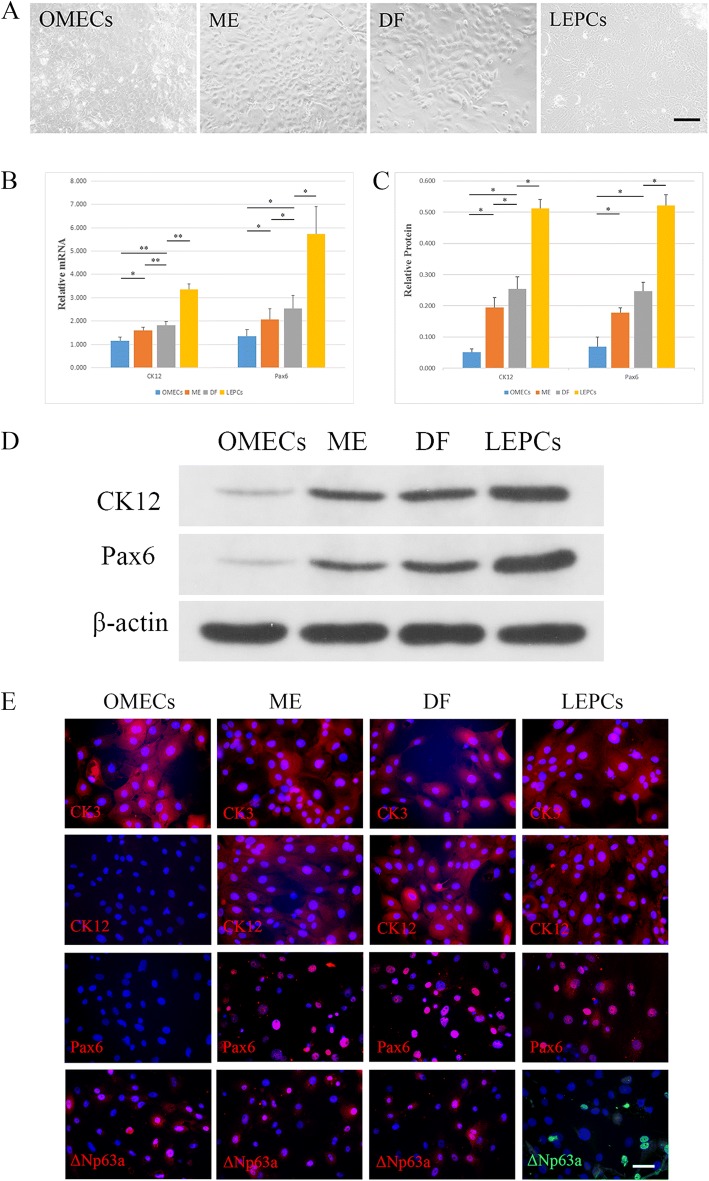
Transwell coculturing of oral mucosal epithelial cells (OMECs) and limbal niche cells (LNCs) in supplemented hormonal epithelial medium (SHEM). **a** Morphologic characterization of OMECs and LNCs cocultured in Transwell. Scale bar = 100 μm. **b** The relative mRNA expression of CK12 and Pax6 in cocultured OMECs and LNCs. **c** The relative protein levels of cocultured OMECs and LNCs. **d** Western blot analysis of CK12 and Pax6 expression in cocultured OMECs and LNCs; β-actin was used as an internal control. **e** Cells were single-stained for CK3, CK12, Pax6, and ΔNp63α, and nuclei were stained by DAPI. Scale bar = 50 μm. **p < 0.01, *p < 0.05. DF Cocultured OMECs supported by DF-LNCs in Transwell, LEPC Limbal epithelial progenitor cell, ME Cocultured OMECs supported by ME-LNCs in Transwell

According to the results of 3D coculturing, we chose SHEM as the culture medium for further experiments. However, the LNCs were still obtained by culture in MESCM or DMEM/F12 supplemented with 10% fetal bovine serum. OMECs cultured in the Transwell system reached confluence at days 10–14. The morphology of OMECs and LEPCs revealed typical slabstone-like features with or without the support of LNCs (Fig. 5a). RT-PCR showed that the transcript levels of CK12 and Pax6 were upregulated for both ME and DF when compared with that of OMECs (Fig. 5b). The transcript level of CK12 in ME and DF was upregulated by 1.590 ± 0.139-fold (*p* = 0.01650, *n* = 3) and 1.817 ± 0.162-fold (*p* = 0.00556, *n* = 3) compared with that in OMECs at 1.158 ± 0.146. The transcript level of CK12 in DF was even higher than that in ME (*p* = 0.00609, *n* = 3) and was statistically different compared with that in LEPCs (3.346 ± 0.246). The transcript level of Pax6 in ME and DF was upregulated by 2.072 ± 0.451-fold (*p* = 0.02621, *n* = 3) and 2.542 ± 0.542-fold (*p* = 0.01419, *n* = 3) compared with that in OMECs at 1.336 ± 0.294. The transcript level of *Pax6* in DF was even higher than that in ME (*p* = 0.04070, *n* = 3) and was also statistically different compared with that in LEPCs (*p* = 0.01312, *n* = 3). The protein level of CK12 in DF was higher when compared with that in OMECs and ME (*p* = 0.01934 compared with OMECs, *p* = 0.01487 compared with ME, *n* = 3) but lower compared with that in LEPCs (*p* = 0.01034, *n* = 3). CK12 levels were also higher in ME than in OMECs (*p* = 0.02748, *n* = 3). The protein level of Pax6 in DF was higher when compared with that in OMECs (*p* = 0.03333, *n* = 3) and lower than that in LEPCs (*p* = 0.01626, *n* = 3) but was not statistically different from that in ME (*p* = 0.05273, *n* = 3). ME also had a higher Pax6 protein level compared with that in OMECs (*p* = 0.04809, *n* = 3). The relative protein levels and results of Western blot assay are shown in Fig. 5c and d, respectively. The immunofluorescence results demonstrated that all groups contained CK3^+^ cells, while the cultured OMECs showed almost negative expression of CK12 and Pax6. On the other hand, LEPCs comprised almost all CK12^+^ and Pax6^+^ cells. ME and DF showed pronounced CK12 and Pax6 expression in a portion of cells, according to the morphologic characterization. We also observed that ME and DF retained some ΔNp63α^+^ cells, as did OMECs and LEPCs. The immunofluorescence of Transwell cocultured groups is shown in Fig. 5e, and additional data are included in Additional file 1: Files S8 and S9.

### Renewing LNCs in the Transwell coculture system and comparison with 3T3 cells

**Fig. 6:**
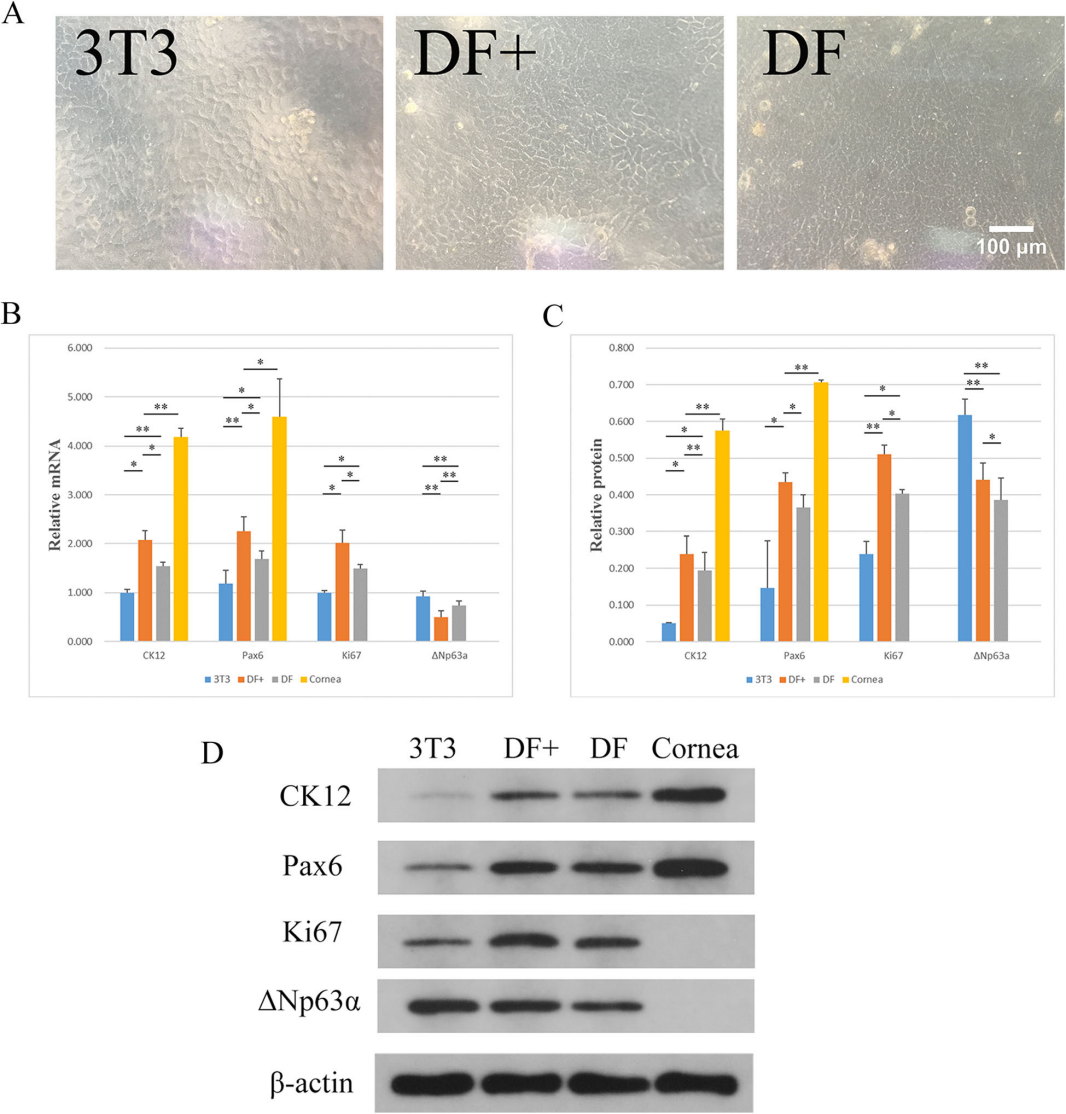
Renewing the DF-LNCs in the Transwell system and comparison with 3T3 cells. **a** Morphologic characterization of OMECs supported by 3T3 cells (3T3), DF-LNC (DF), and renewed DF-LNC (DF+) in Transwell. Scale bar = 100 μm. **b** The relative mRNA expression of CK12, Pax6, Ki67, and ΔNp63α expressed by OMECs supported by 3T3 cells, DF-LNC, and renewed DF-LNC in Transwell. **c** The relative protein level of CK12, Pax6, Ki67, and ΔNp63α expressed by OMECs supported by 3T3 cells, DF-LNC, and renewed DF-LNC in Transwell. **d** Western blot analysis of CK12, Pax6, Ki67, and ΔNp63α; β-actin was used as an internal control. **p < 0.01, *p < 0.05

After comparing the effect of transdifferentiation in the Transwell system that was supported by ME-LNC or DF-LNC, we chose DF-LNC as the feeder layer for further experiments, while 3T3 cells were used as a control. LNCs cocultured with OMECs in all groups showed a typical slabstone-like morphology (Fig. 6a). The transcript level of CK12 was upregulated by 2.080 ± 0.183-fold and 1.542 ± 0.086-fold in the DF+ and DF groups, respectively, when compared with that in the 3T3 group (*p* < 0.05, *n* = 3). DF+ was also statistically different when compared with DF and the cornea (all *p* < 0.05, *n* = 3). The transcript level of Pax6 was upregulated by 2.255 ± 0.289-fold and 1.681 ± 0.166-fold in the DF+ and DF groups, respectively, when compared with that in the 3T3 group (*p* < 0.05, *n* = 3). Furthermore, the expression level of Pax6 in DF+ was statistically different from that in DF and the cornea (all *p* < 0.05, *n* = 3). The relative mRNA level of Ki67 was upregulated by 2.010 ± 0.262-fold and 1.488 ± 0.088-fold in the DF+ and DF groups, respectively, compared with that in the 3T3 group (*p* < 0.05, *n* = 3). Specifically, Ki67 expression was most pronounced in the DF+ group. Moreover, the expression level of ΔNp63α was highest in the 3T3 group when compared with that in the DF+ and DF groups (*p* < 0.01, *n* = 3), while DF+ showed the lowest expression of ΔNp63α (Fig. 6b). Western blot showed similar results regarding protein expression of CK12, Pax6, and Ki67, except there were no statistically significant differences between 3T3 and DF at the protein level of Pax6 (*p* = 0.06595, *n* = 3) (Fig. 6c, d). In conflict with the results of RT-PCR, DF+ was higher than the DF group at the protein level for ΔNp63α (*p* = 0.02418, *n* = 3), though they were both statistically significant different from 3T3 (*p* < 0.01, *n* = 3). Further data and analysis are included in Additional file 1: Files S10 and S11.

### Airlift culture

**Fig. 7:**
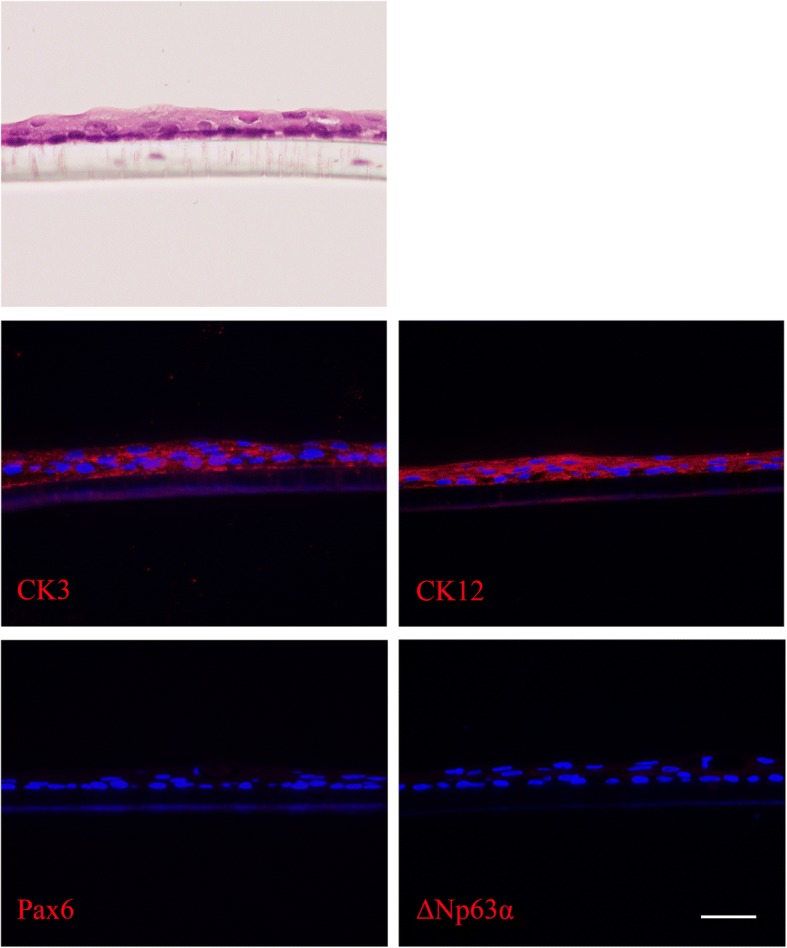
Hematoxylin and eosin staining and immunofluorescence of airlift cocultured OMECs. Cocultured OMECs were stained with hematoxylin and eosin and single-stained for CK3, CK12, Pax6, and ΔNp63α. Scale bar = 100 μm

The hematoxylin and eosin staining of airlift cocultured OMECs showed stratified structures for 2–3 layers. The entire layer of the stratified epithelium expressed CK3 and CK12; however, this was negative for Pax6 and ΔNp63α (Fig. 7).

**Fig. 8:**
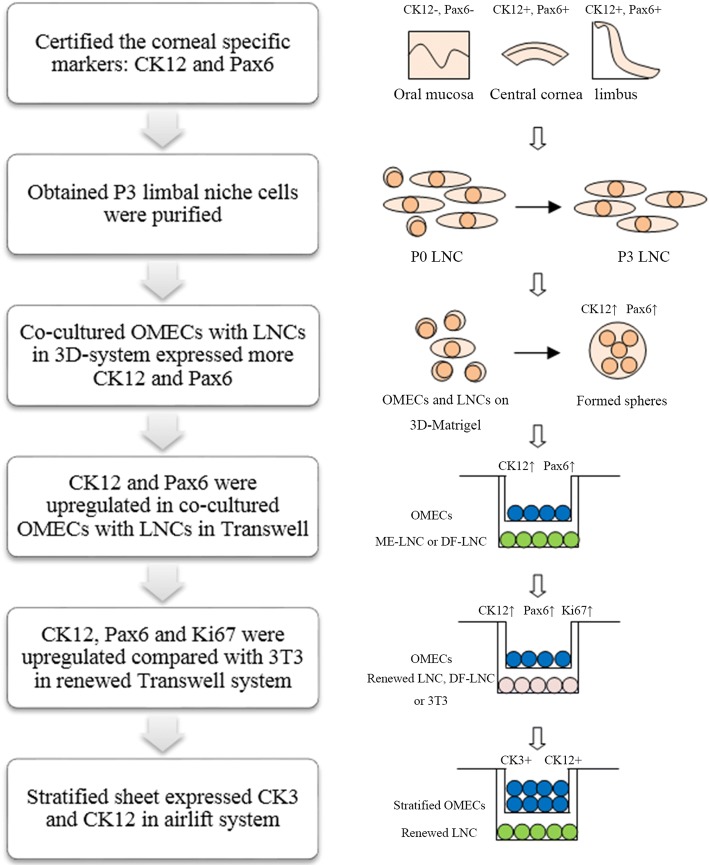
Flow chart describing the steps involved in confirmation of transdifferentiation of OMECs. This flowchart shows the logic flow behind arriving at the results that we have presented and the conclusions drawn from those results. DF-LNC LNCs grown in DMEM/F12 + 10% FBS, LNC limbal niche cell, ME-LNC LNCs grown in MESCM, OMEC oral mucosal epithelial cell

A flowchart describing the steps involved in confirmation of transdifferentiation of OMECs is provided in Fig. 8.

## Discussion

In this study, we achieved the transdifferentiation of rat OMECs into corneal epithelial-like cells with the help of LNCs in vitro. Moreover, we demonstrated that LNCs as well as 3T3 cells have the ability to support OMECs.

At the beginning of our study, we evaluated the immunofluorescence of the oral mucosa, central cornea, and limbus to prove that only CK12 and Pax6 could be defined as cornea-specific markers, as previously reported [26–28]. We agree with previous studies [23] suggesting that CK3 cannot be considered a cornea-specific marker, and CK3/12 is a marker of epithelium differentiation. Furthermore, we also confirmed that oral mucosa and limbus express the same stem cell marker, ΔNp63α, as previously described [29].

LNCs have been involved in many studies; these cells are localized in the limbus subjacent to the epithelial basement membrane and maintain a close association with limbal epithelial stem cells [20, 21, 25, 30]. Several methods to isolate LNCs have been described [20, 25, 31], but the current consensus is that digestion of limbal tissue with collagenase results in optimal LNCs [32]. Vimentin was the first marker identified at the base of the limbus [33]. Oct4 plays an important role in maintaining the pluripotency and self-renewal of embryonic stem cells, and it has been proven to be expressed in the basal limbus [34]. Sox2 is also considered a marker of LNCs [35], and N-cadherin has been detected in LNCs and melanocytes [36]. Therefore, we performed double immunofluorescence staining on P3 LNCs to confirm that we had obtained purified cells, which represented the phenotype of limbal niche cells*,* cultured in either MESCM or DMEM/F12 supplemented with 10% fetal bovine serum. As a result, LNCs did not interfere with the results of further coculture. The results of RT-PCR and Western blotting regarding Oct4 and Sox2 expression in LNCs indicated that using MESCM for culturing rather than DMEM/F12 supplemented with 10% fetal bovine serum could produce LNCs that expressed more mesenchymal stem cell markers, as previously reported [20].

Three-dimensional cocultured OMECs and LNCs produced spheres owing to the 3D Matrigel [37], and other studies have confirmed that LNCs have the ability to attract and aggregate the epithelium [20, 21]. Results of 3D coculturing demonstrated that use of SHEM and DF-LNCs could upregulate the expression of CK12 and Pax6, indicating that they are better for transdifferentiation of OMECs to corneal epithelial-like cells. However, MESCM is not suitable for transdifferentiation. We consider these results to be due to the ability of MESCM to maintain the phenotype of stem cells and prevent their differentiation [20, 21, 25, 38]. Furthermore, we also demonstrated that maintaining the phenotype of LNCs does not benefit transdifferentiation. We consequently attempted to coculture OMECs and LNCs in the Transwell system to obtain a transplantable epithelium sheet. MESCM failed to support the growth of OMECs in the early period of the study, forcing us to abandon this medium in the Transwell system. When we compared the transdifferentiation effect of ME-LNCs and DF-LNCs in the Transwell system, we observed results similar to those obtained with the 3D coculturing system, showing that DF-LNCs were more effective than ME-LNCs. Immunofluorescence assay of cultured OMECs, ME, DF, and LEPCs confirmed that CK3 cannot be defined as a cornea-specific marker, whereas higher expression levels of CK12 and Pax6 confirmed the transdifferentiation of OMECs into corneal epithelial-like cells after coculturing with ME-LNCs or DF-LNCs. Moreover, we noticed that some ΔNp63α^+^ cells remained after coculturing, prompting us to compare the OMEC support by LNCs and 3T3 cells, which are considered the gold standard for the feeder layer. At the same time, we attempted to renew the LNCs in the culture system. 3T3 cells are considered the gold standard for the feeder layer because of their ability to support the proliferation and maintain the phenotype of OMECs [9, 10, 12]. The expression level of CK12, Pax6, and Ki67 was also upregulated when compared with that in other groups by renewing the LNCs in the Transwell coculturing system, suggesting that this might be a potential method for improving the efficiency of transdifferentiation and proliferation of OMECs. However, ΔNp63α expression was different between the DF+ and DF groups at both the transcript and protein level, although it was most pronounced in the 3T3 group. We considered this outcome the effect of complex molecular mechanisms in transdifferentiation, which requires further study. The obtained stratified cocultured OMECs showed 2–3 layers and expressed CK3 and CK12, indicating that they were well differentiated, and had a corneal epithelial-like phenotype, which might be a potential source for ocular reconstruction. In conflict with a previous study [16], Pax6 and ΔNp63α were negative, probably because of long-term culturing by the airlifting method [28, 37], although they were both pronounced when cocultured OMECs reached confluence.

We also noticed that 3T3 cocultured OMECs did not show as much negative expression, especially for Pax6, as cultured OMECs with no feeder layer. Other researchers have encountered a similar situation [23]; however, no evidence has indicated that 3T3 cells cocultured with OMECs can upregulate the expression of Pax6. Even so, coculturing with LNCs on a 3D Matrigel or in the Transwell system upregulated the expression of CK12 and Pax6 when compared with that in OMECs alone or cocultured with 3T3, which conflicts with a previous study [23]. We believe that the culturing system and method of LNC acquisition are key determiners. In the previous study, the researchers used a direct-contact coculture system in which OMECs were merged with limbal fibroblast cells, and did not obtain positive results. In this direct-contact system, feeder cells were very difficult to exchange, although the researchers described a T/E digestion method in which the OMECs would be trypsinized every time they changed the feeder layers. To our understanding, other literature focusing on adult stem cell transdifferentiation into corneal epithelial-like cells is based on a limbal niche environment offered by conditioned medium, which consisted of the medium that had been used for limbal niche cell culture [15, 18, 19]. We improved this method using the Transwell system, which was loaded with fresh conditioned medium and not frozen. This 0.4-μm Transwell system can reliably prevent cells in the lower chamber from entering the upper chamber [39]. The 3D Matrigel coculture system is a very advanced coculturing system used to investigate intercellular signaling and pathways that can simulate the niche microenvironment in vitro [21, 30]. Although we merged OMECs and LNCs in this system, we verified that we obtained purified LNCs that would not produce interference, and LNCs in this system did not require changing. Furthermore, the obtained niche cells were different from limbal fibroblast cells as previously described [31]. Other researchers have used a special matrix [40] or factors [41] to achieve transdifferentiation of OMECs into corneal-like epithelium, support for which we believe requires further evidence. Although we succeeded in transdifferentiating rat OMECs into corneal epithelial-like cells in vitro, whether the same can occur in human cells is unknown and will be investigated by us in the future.

Very recently, keratoplasty lenticules, a novel carrier, have been proven to be suitable for transplantation to reconstruct the ocular surface and treat LSCD [42]. Our research team has succeeded in culturing OMECs on acellular porcine corneal stroma (APCS) [24], and a clinical trial in our hospital has demonstrated that APCS transplantation is useful for treating fungal keratitis [43]. Based on these investigations, we are also attempting to coculture OMECs on APCS with LNCs as feeder layers in a Transwell system for further transplantation in animal experiments (Additional file 1: File S12). This treatment method appears promising for the treatment of LSCD in the future.

## Conclusion

In summary, we achieved transdifferentiation of OMECs into corneal epithelial-like cells in vitro. We evaluated two coculturing systems: 3D Matrigel for investigation of signaling pathways between OMECs and LNCs and a Transwell system for culturing a transplantable epithelium sheet. These cells have the potential to serve as an alternative source for transplantation. This investigation is of great significance for the treatment of LSCD and ocular surface reconstruction.

## Additional file

Additional file 1: **S1.** The materials used in cell isolation and culture. **S2.** The primer sequences used in RT-PCR. **S3.** The antibodies used in the experiments. **S4.** PCR of DF-LNC and ME-LNC. **S5.** Western blot of DF-LNC and ME-LNC. **S6.** PCR of 3D cocultured OMECs and LNCs. **S7.** Cell counting of 3D cocultured OMECs and LNCs. **S8.** PCR of Transwell cultured groups. **S9.** Western blot of Transwell cultured groups. **S10.** PCR of 3T3 cells and renewed LNCs cultured in Transwell. **S11.** Western blot of 3T3 cells and renewed LNCs cultured in Transwell. **S12.** Cocultured OMECs on APCS. **S13.** IACUC. (ZIP 6676 kb)

## Abbreviations

APCS: Acellular porcine corneal stroma
DF+: Cocultured OMECs supported by renewed DF-LNCs in Transwell
DF: Cocultured OMECs supported by DF-LNCs in Transwell
DF-LNCs: LNCs grown in DMEM/F12 supplemented with 10% fetal bovine serum
DF-ME: DF-LNCs 3D cocultured in MESCM
DF-SH: DF-LNCs 3D cocultured in SHEM
DMEM/F12: Dulbecco’s modified Eagle’s medium
LEPC: Limbal epithelial progenitor cell
LNC: Limbal niche cell
LSCD: Limbal stem cell deficiency
ME: Cocultured OMECs supported by ME-LNCs in Transwell
ME-LNCs: LNCs grown in MESCM
ME-ME: ME-LNCs 3D cocultured in MESCM
MESCM: Modified embryonic stem cell medium
ME-SH: ME-LNCs 3D cocultured in SHEM
OMEC: Oral mucosal epithelial cell
PBS: Phosphate-buffered saline
RT-PCR: Reverse-transcription polymerase chain reaction
SHEM: Supplemented hormonal epithelial medium
T/E: 0.25% trypsin/0.02% ethylenediaminetetraacetic acid
