# Correction to: Rat limbal niche cells can induce transdifferentiation of oral mucosal epithelial cells into corneal epithelial-like cells in vitro

**DOI:** 10.1186/s13287-018-1040-9

**Published:** 2018-10-25

**Authors:** X. Y. Zhao, H. T. Xie, C. Y. Duan, J. Li, M. C. Zhang

**Affiliations:** 1 Department of Ophthalmology, Union Hospital, Tongji Medical College, Huazhong University of Science and Technology, Wuhan, 430022 China; 2 Department of Ophthalmology, Tongji Hospital, Tongji Medical College, Huazhong University of Science and Technology, Wuhan, 430022 China

## Correction

The original article [1] contains an error in Fig. 5e whereby the immunofluorescence of ΔNp63α in the ME group is incorrectly presented; thus, the corrected figure is shown ahead.

**Fig. 5:**
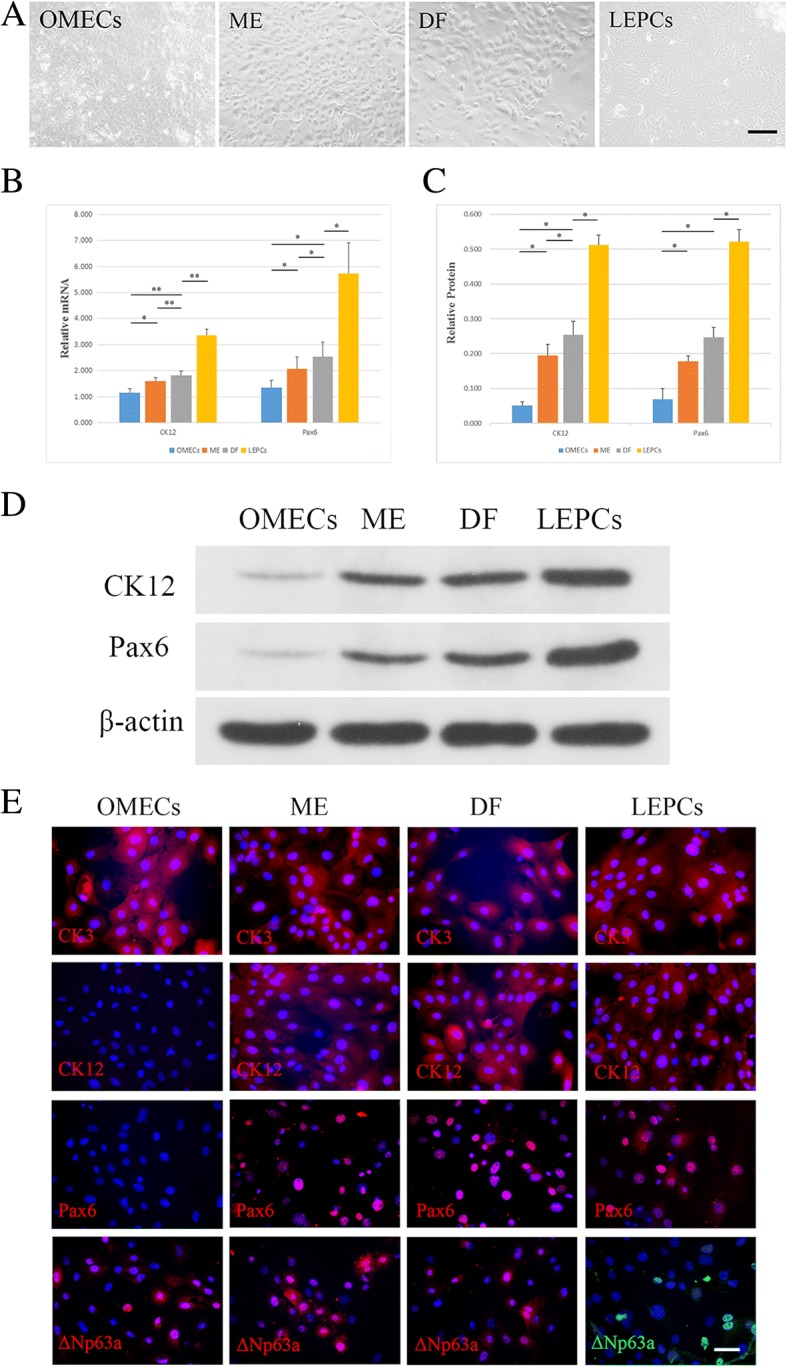
Transwell coculturing of oral mucosal epithelial cells (OMECs) and limbal niche cells (LNCs) in supplemented hormonal epithelial medium (SHEM). **a** Morphologic characterization of OMECs and LNCs cocultured in Transwell. Scale bar = 100 μm. **b** The relative mRNA expression of CK12 and Pax6 in cocultured OMECs and LNCs. **c** The relative protein levels of cocultured OMECs and LNCs. **d** Western blot analysis of CK12 and Pax6 expression in cocultured OMECs and LNCs; β-actin was used as an internal control. **e** Cells were single-stained for CK3, CK12, Pax6, and ΔNp63α, and nuclei were stained by DAPI. Scale bar = 50 μm. **p < 0.01, *p < 0.05. DF Cocultured OMECs supported by DF-LNCs in Transwell, LEPC Limbal epithelial progenitor cell, ME Cocultured OMECs supported by ME-LNCs in Transwell
