# Supplementary material for: Rat limbal niche cells can induce transdifferentiation of oral mucosal epithelial cells into corneal epithelial-like cells in vitro
Source: Stem Cell Res Ther. 2018 Sep 26;9:256. doi: 10.1186/s13287-018-0996-9 (PMC6158850; doi:10.1186/s13287-018-0996-9)
Supplement: Supplementary file 1 — S1. The materials used in cell isolation and culture. S2. The primer sequences used in RT-PCR. S3. The antibodies used in the experiments. S4. PCR of DF-LNC and ME-LNC. S5. Western blot of DF-LNC and ME-LNC. S6. PCR of 3D cocultured OMECs and LNCs. S7. Cell counting of 3D cocultured OMECs and LNCs. S8. PCR of Transwell cultured groups. S9. Western blot of Transwell cultured groups. S10. PCR of 3T3 cells and renewed LNCs cultured in Transwell. S11. Western blot of 3T3 cells and renewed LNCs cultured in Transwell. S12. Cocultured OMECs on APCS. S13. IACUC. (ZIP 6676 kb) [file 13287_2018_996_MOESM1_ESM.zip › S13 IACUC.pdf]

地址：武汉市航空路 13 号　邮编：430030
Address: 13 Hangkong Road, Wuhan, 430030
电话和传真：027-83692751
Telephone & Fax: 027-83692751

华中科技大学同济医学院实验动物伦理委员会审批报告【只填英文】
The Institutional Animal Care and Use Committee at Tongji Medical College, Huazhong University of Science and Technology

[2016] 伦审字 （S760）号
[2016] IACUC Number: S760

<table>
<tr><td>项目名称
Title of Project</td><td colspan="3">Rat Limbal Niche Cells Can Induce Transdifferentiation of Oral Mucosal Epithelial Cells into Corneal Epithelial-like Cells In Vitro</td></tr>
<tr><td>申请单位
Application Institute</td><td colspan="3">Department of Ophthalmology, Union Hospital, Tongji Medical College, Huazhong University of Science and Technology</td></tr>
<tr><td>项目负责人
Principal Investigator</td><td>Ming-Chang Zhang</td><td>职称
Title</td><td>Professor</td></tr>
<tr><td rowspan="3">报 送 资 料
Material submitted</td><td colspan="2">课题研究方案
Research Plan</td><td>有■　无□
Yes ■　No □</td></tr>
<tr><td colspan="2">观察记录表
Observational Recording Table</td><td>有■　无□
Yes ■　No □</td></tr>
<tr><td colspan="2">研究人员名单
List of Researchers</td><td>有■　无□
Yes ■　No □</td></tr>
<tr><td rowspan="2">审 查
Censor</td><td colspan="2">研究者资格
Qulification of Researchers</td><td>符合要求■　不符合要求□
Meet ■　Not meet □</td></tr>
<tr><td colspan="2">课题研究方案
Research Plan</td><td>适当■　不适当□
Appropriate ■　Not appropriate □</td></tr>
<tr><td>有效期
Period of Validity</td><td colspan="3">September 9, 2016 - May 2, 2018</td></tr>
</table>

审评意见：
Remarks:

本伦理委员会审阅并讨论了上述相关资料，该课题研究符合《湖北省实验动物管理条例》和《华中科技大学同济医学院实验动物伦理委员会章程》，经伦理委员会审核，同意该课题实施。

The Tongji Medical College, HUST Institutional Animal Care and Use Committee has reviewed and discussed the above metioned materials, and accepted the proposal and approve the research plan as described

华中科技大学同济医学院实验动物伦理委员会
Institutional Animal Care and Use Committee
Tongji Medical College, Huazhong University of Science and Technology

负责人签字：[signature]
Signiture:

批准日期：2016年9月9日
Date: Sep. 9th, 2016
