# Supplementary figures and images for: Rat limbal niche cells can induce transdifferentiation of oral mucosal epithelial cells into corneal epithelial-like cells in vitro
Source: Stem Cell Res Ther. 2018 Sep 26;9:256. doi: 10.1186/s13287-018-0996-9 (PMC6158850; doi:10.1186/s13287-018-0996-9)

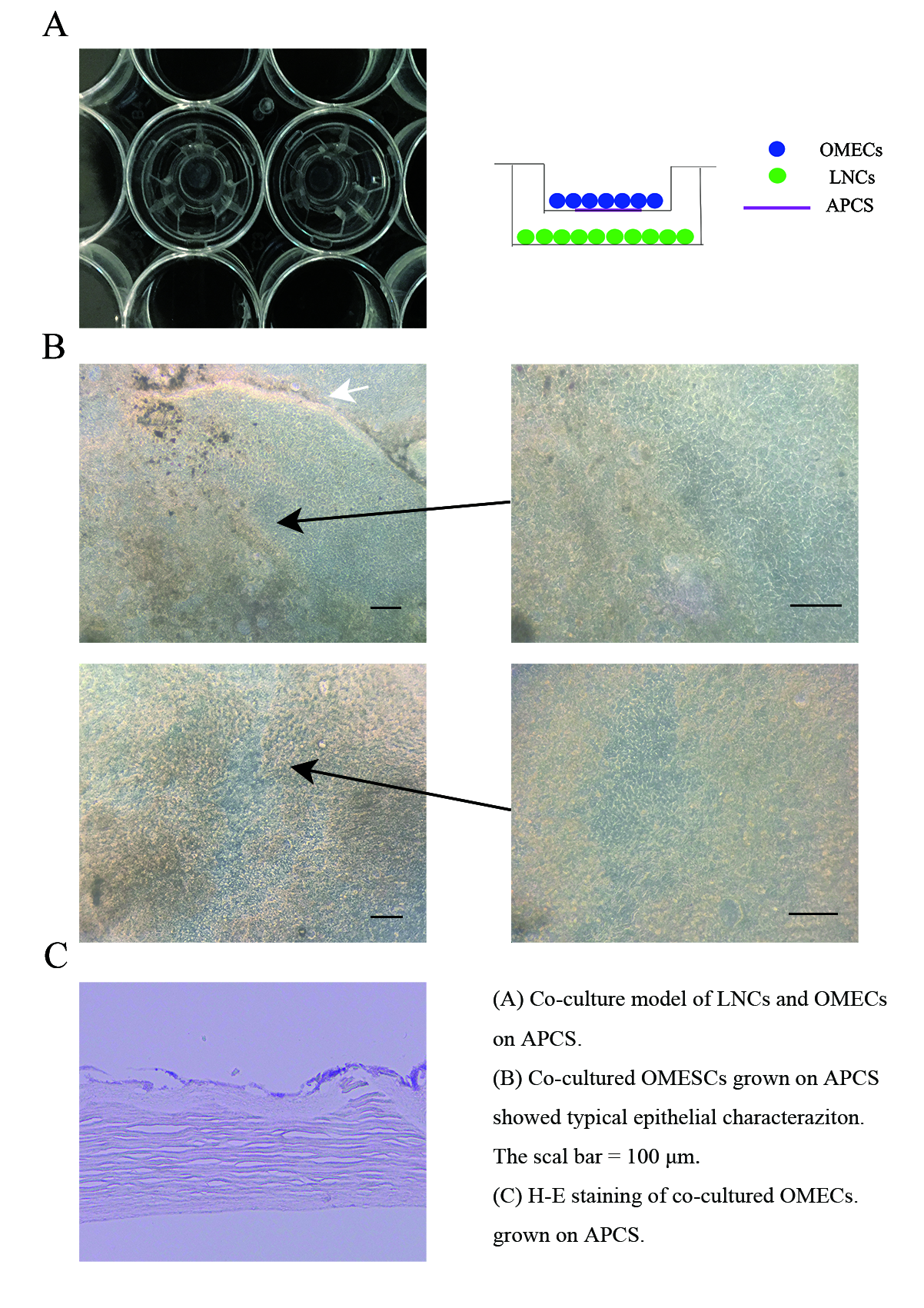

Supplement: Supplementary file 1 — S1. The materials used in cell isolation and culture. S2. The primer sequences used in RT-PCR. S3. The antibodies used in the experiments. S4. PCR of DF-LNC and ME-LNC. S5. Western blot of DF-LNC and ME-LNC. S6. PCR of 3D cocultured OMECs and LNCs. S7. Cell counting of 3D cocultured OMECs and LNCs. S8. PCR of Transwell cultured groups. S9. Western blot of Transwell cultured groups. S10. PCR of 3T3 cells and renewed LNCs cultured in Transwell. S11. Western blot of 3T3 cells and renewed LNCs cultured in Transwell. S12. Cocultured OMECs on APCS. S13. IACUC. (ZIP 6676 kb) [file 13287_2018_996_MOESM1_ESM.zip › S12 Co-cultured OMECs on APCS.tif]
